# Latent profile and associated factors of digital health literacy among patients with coronary heart disease: a cross-sectional study

**DOI:** 10.3389/fpubh.2026.1844333

**Published:** 2026-05-26

**Authors:** Lan Luo, Tingting Liao, Yanmei Gan, Mengjia Yi, Baolin Zou, Yaoqiong Lu, Yunxu Huang, Jie Liang, Gaoye Li

**Affiliations:** 1 China-ASEAN Geriatric Cardiology Division, First Affiliated Hospital of Guangxi Medical University, Nanning, Guangxi, China; 2 China-ASEAN Cardiovascular Medicine Department I, First Affiliated Hospital of Guangxi Medical University, Nanning, Guangxi, China

**Keywords:** associated factors, China, coronary heart disease, digital health literacy, latent profile analysis, nursing

## Abstract

**Objective:**

This study aims to explore the potential characteristics and associated factors of digital health literacy among patients with coronary heart disease, and to guide targeted intervention measures.

**Methods:**

This study used convenience sampling to recruit 341 patients with coronary heart disease from a tertiary hospital in the Guangxi region from March to May 2025. Data were collected using a general information questionnaire, the Chinese version of the Digital Health Literacy Scale, the Health Belief Model Scale, and the Social Support Rating Scale. Latent profile analysis (LPA) was conducted using Mplus 8.3 to identify latent profiles of digital health literacy. Subsequently, univariate and multinomial logistic regression analyses were performed using SPSS 27.0 to explore factors associated with the identified profiles.

**Results:**

Among the 341 patients with coronary heart disease, the mean age was 63.38 years (SD = 9.96). The mean digital health literacy score was 19.27 (SD = 9.27), indicating a relatively low level. LPA identified three distinct profiles: C1-Low Literacy, Passive Reception Type (48.1%); C2-Moderate Literacy, Application but Poor Appraisal Type (31.7%); and C3-High Literacy, Autonomous Decision-Making Type (20.2%). Multinomial logistic regression analysis revealed that older age was associated with lower digital health literacy, while higher education level, urban residence, greater social support, and stronger health beliefs were associated with higher digital health literacy profiles (all *p* < 0.05).

**Conclusion:**

Digital health literacy among patients with coronary heart disease was generally low and exhibited significant heterogeneity across profiles. Healthcare providers should develop tailored interventions based on the characteristics of different subgroups to improve digital health literacy in this population.

## 1 Introduction

Coronary heart disease (CHD), also known as coronary atherosclerotic heart disease, is characterized by high prevalence, high mortality, high readmission rates, and frequent hospitalizations (1). In 2018, there were approximately 110.6 million CHD patients worldwide, and the prevalence of CHD is projected to increase to 18.0% by 2030 (2). The China Cardiovascular Health and Disease Report 2024 (1) indicated that approximately 330 million people in China live with cardiovascular disease, with CHD affecting about 11.39 million individuals, ranking first among cardiovascular diseases. China has now become a moderately aging society, with the incidence and mortality of CHD rising continuously over the past decade (3). CHD has now become the leading cause of death in six provinces and cities in China, making it the most prevalent chronic disease in the country (4). This imposes a heavy economic and disease burden on individuals, families, and society (1). Therefore, effective control and self-management of CHD should be a collective task undertaken by modern society.

As digitalization accelerates, digital health governance has received growing attention (5). China's healthcare system is rapidly transforming toward digital health to address the increasing demand for digital health services (5). With ongoing innovation and development in digital health technologies, they are increasingly being applied to self-health management, including real-time monitoring, online health consultations, empowerment of medical decision-making, digital care, personalized feedback, and behavioral adjustment (6–8). These services enhance the accessibility of health resources for patients with coronary heart disease, promote the adoption of a healthy lifestyle, and contribute to improved disease prognosis (9).

Digital health literacy (DHL) is increasingly being recognized as a critical determinant of both public and individual health (10). DHL is defined as an individual's ability to search for, obtain, evaluate, and apply health information through digital technology, participate in online exchanges and interactions related to health information, and use the obtained information for health management and problem-solving (11). Exposure to unverified or poor-quality health information may negatively affect patients' understanding of their disease (12). Contradictory or misleading medical content can easily cause confusion in treatment decisions, weaken patients' trust in healthcare providers, and intensify their sense of social isolation (13). In addition, prolonged exposure to negative health information may cause algorithms to further recommend similar distressing content. Therefore, enhancing the DHL of CHD patients is essential. Individuals with higher DHL are better able to access evidence-based cardiac rehabilitation strategies, psychological support resources, and channels for connecting with patient communities (14). These resources help patients gain a more comprehensive understanding of disease prognosis, reducing anxiety and uncertainty about disease progression.

Social support refers to the comfort, help, or information that an individual receives from others. In offline settings, social support mainly comes from family and friends, whereas in online settings, social media has become an important channel for patients to obtain social support (15). According to social cognitive theory, social support is considered a fundamental determinant of patients' acquisition of digital health knowledge (15, 16). Shao Yujiao (17) confirmed that social support significantly influences DHL, and the availability and quality of social support may vary considerably across different patient subgroups. Strong social support from family members, peers, or healthcare providers, including emotional encouragement, informational guidance, and tangible assistance (18, 19), enhances patients' confidence and capacity to utilize digital health resources. This study employed the Social Support Rating Scale to assess social support, which emphasizes subjective support, objective support, and the utilization of support individuals receive from their social network (20).

Health beliefs refer to an individual's views and attitudes toward health behaviors, which shape their health-related behavioral decisions (21). The Health Belief Model (HBM) provides an important theoretical perspective for further understanding the formation mechanism of DHL among patients with coronary heart disease. The Health Belief Model posits that an individual's perception of health risks (such as perceived susceptibility and perceived severity) and the benefits of health behaviors (including perceived benefits, perceived barriers, and self-efficacy) guide their health-related decision-making (22). Individuals with a high level of health awareness are more likely to actively seek and attend to health-related information to meet their health information needs (23). People with higher DHL are better able to obtain, interpret, and apply health information, such as understanding the link between health outcomes and behaviors and adjusting their beliefs and actions accordingly (24). Studies have shown that enhancing confidence in mental health self-management promotes positive self-help behaviors among patients (25). Health beliefs may influence the DHL of CHD patients through multiple pathways. Specifically, patients with a stronger perception of CHD risk are more likely to actively search for online health information; those who recognize the value of digital health tools are more willing to use them; and those who are confident in their abilities are better able to master and apply digital health skills. Therefore, exploring the relationship between health beliefs and DHL among CHD patients is of great significance for revealing the underlying formation mechanisms and developing targeted intervention strategies.

The importance of DHL is increasingly recognized. However, existing studies have predominantly focused on students, older adults, community residents, and individuals with chronic conditions such as hypertension (26–28). Although research on cardiovascular populations has grown (29, 30), the findings remain inconsistent. Ramstad et al. (29) found that after PCI, patients with higher DHL were more proactive in using digital health resources, including online health information searching and health-related applications, suggesting that DHL may contribute to secondary prevention. The study by Chepkorir et al. (30) was more complex. Higher DHL was associated with more frequent fruit and vegetable consumption and higher sugar-sweetened beverage intake, but no significant association was found between DHL and cardiovascular disease or its risk factors. These inconsistent results indicate that DHL plays an important role in cardiovascular health, yet its impact may vary by population characteristics, individual patient differences, or outcome measures, exhibiting population heterogeneity. Furthermore, research on DHL in patients with CHD remains insufficient. In previous studies on CHD, most have adopted a variable-centered approach, focusing on the current status, correlations, mediating effects, and associated factors of DHL, without fully considering heterogeneous patient groups or the relationships among variables (17, 26, 31, 32). To date, no study has explored the potential population heterogeneity of DHL among CHD patients or its complex relationships with health beliefs and social support. There is also a lack of systematic analysis of the intrinsic factors that may affect this relationship.

Latent Profile Analysis (LPA) is a person-centered approach that classifies individuals into distinct latent subgroups based on their shared response patterns across multiple indicators, thereby revealing group heterogeneity and improving classification accuracy (33). This method enables more precise identification of subgroup characteristics, providing a basis for personalized interventions. Therefore, given the above research gaps and the methodological advantages of LPA, the present study employed LPA to explore the latent profiles of DHL among CHD patients, examine differences in demographic characteristics across profiles, and test their associations with health beliefs and social support. The research questions were: (1) What latent profiles of DHL exist in CHD patients? (2) How do demographic characteristics vary across profiles? (3) Do health beliefs and social support levels differ significantly across profiles? The findings will provide a theoretical basis and empirical support for developing targeted intervention strategies, and will offer new evidence to address the inconsistent findings in the field of digital health literacy in cardiovascular disease.

## 2 Participants and methods

### 2.1 Participants

This study employed convenience sampling. A total of 341 patients with coronary heart disease were recruited from the Department of Cardiology of a tertiary hospital in Guangxi Zhuang Autonomous Region. Data were collected from March 2025 to May 2025. Inclusion criteria: (1) age ≥18 years; (2) diagnosis of coronary heart disease confirmed by coronary angiography or CTA, consistent with the WHO diagnostic criteria (34); (3) ability to communicate normally; (4) informed consent and voluntary participation in this study. Exclusion criteria: (1) severe cognitive impairments or psychiatric disorders; (2) acute or critical illness complicated by severe complications or organ failure during the study period; (3) concurrent participation in other related studies.

This study included 18 demographic variables, the Digital Health Literacy Scale (3 dimensions), the Health Belief Model Scale (5 dimensions), and the Social Support Rating Scale (3 dimensions), totaling 29 predictors (including dummy-coded categories). According to Kendall's sample size estimation method, the sample size should be 5 to 10 times the number of independent variables in a quantitative cross-sectional study (35), estimating the required sample size to be between 145 and 290. Considering a 10% invalid response rate, the final calculated target sample size was *n* = (145–290) × (1 + 10%) = 160–319 cases. Our study also conducted LPA on the DHL of CHD patients. Nylund-Gibson and Choi (36) suggested that the minimum adequate sample size standard for LPA should be 300 cases. Therefore, based on the above criteria and considering the invalid questionnaire rate, we finally included 341 valid samples, meeting the sample size estimation requirements.

### 2.2 Research tools

#### 2.2.1 General information questionnaire

This questionnaire was developed by the research team through an extensive literature review. It collected data on demographics (e.g., age, gender, BMI, educational level, marital status, number of co-residents, place of residence, per capita monthly household income, self-rated economic status, medical insurance type, self-rated health) and disease-related characteristics (e.g., CHD duration, smoking history, drinking history, family history, regular checkups, regular exercise, number of comorbidities).

#### 2.2.2 Digital health literacy scale (eHEALS)

The eHEALS was originally developed by Norman et al. (37) and later translated and adapted for the Chinese context by Guo et al. (38), who validated its psychometric properties in a sample of high school students. The validation study reported a Cronbach's α of 0.913 and factor loadings ranging from 0.692 to 0.869, indicating good reliability and validity, and retained the three-dimensional structure with eight items: ability to apply online health information and services (items 1–5), critical evaluation ability (items 6–7), and health decision-making ability (item 8) (38). Although some studies have reported a unidimensional structure, this study adopted the three-dimensional structure because it is consistent with the theoretical framework of digital health literacy, and this three-dimensional structure has been validated in Chinese populations (32, 38). Each item is scored on a 5-point Likert scale ranging from 1 (“strongly disagree”) to 5 (“strongly agree”), with total scores ranging from 8 to 40; higher scores indicate higher levels of digital health literacy (37, 38). In this study, the scale demonstrated excellent internal consistency, with a Cronbach's α coefficient of 0.946.

#### 2.2.3 Health beliefs scale (HBS)

The Health Belief Scale used in this study was adapted by Ji et al. (39) from the Chinese version of the Nursing Outcomes Classification through a cross-cultural adaptation process, resulting in a version suitable for the Chinese population. The original scale consists of two parts; the present study used the second part, i.e., the Health Belief Scale. The scale comprises 48 items across five dimensions: personal health beliefs (10 items), perceived capability for implementation (7 items), perceived control (6 items), perceived resource utilization (14 items), and perceived threat (11 items) (39). Each item is rated on a 5-point Likert scale (1 = very weak, 5 = very strong). The total score is the sum of all items (range 48–240), with higher scores indicating stronger health beliefs (39). Regarding construct validity, the scale showed a KMO value of 0.901, a statistically significant Bartlett's test of sphericity (*p* < 0.05), and a cumulative variance contribution rate of 54.993% (39). The scale also demonstrated good reliability, with a Cronbach's α of 0.94 and a split-half reliability of 0.94 (39). This scale has been applied in Chinese patients with chronic diseases and is therefore suitable for the CHD patients in our study (40). In the present study, the scale showed excellent internal consistency, with a Cronbach's α coefficient of 0.931.

#### 2.2.4 Social support rating scale (SSRS)

The Social Support Rating Scale, revised by Xiao Shuiyuan (20) in 1994, was used to assess individuals' levels of social support. The scale comprises three dimensions and 10 items: objective support (3 items), subjective support (4 items), and utilization of support (3 items) (20). Scoring is as follows: items 1–5 and 8–11 are single-choice questions scored from 1 to 4; for items 6–7, a score of 0 is assigned if no source is identified, with the total score representing the number of sources reported. The total score ranges from 8 to 66, with higher scores indicating greater social support. A score of ≤ 22 indicates low social support, 23–44 indicates moderate social support, and ≥45 indicates high social support (20). In this study, the Cronbach's α coefficient for the scale was 0.815.

### 2.3 Data collection

This study was approved by the relevant hospital ethics committee and administrative departments. Trained clinical nurses or nursing postgraduate students collected the survey data. The questionnaire was administered within 24 to 48 h of hospitalization, when patients' conditions were stable. Prior to the survey, patients were informed of the research purpose, procedures, and measures to ensure confidentiality and anonymity. They were also assured that participation was voluntary and would not affect their medical care. All patients provided written informed consent. The survey was administered in a quiet ward setting. Patients completed the paper-based questionnaire independently. For those with visual, reading, writing, or physical difficulties, researchers provided one-on-one assistance by interpreting the questions without influencing the responses. Disease-related information was obtained from electronic medical records. To minimize potential bias, researchers assisted only with questionnaire delivery and interpretation. After collection, all questionnaires were anonymized to protect participant privacy. A total of 350 patients with coronary heart disease who met the inclusion criteria were invited to participate. Questionnaires were reviewed on-site, and missing or ambiguous responses were verified immediately. Ultimately, 341 valid questionnaires were obtained, yielding an effective response rate of 97.43%. Nine invalid questionnaires were excluded (six with patterned responses and three with logical contradictions), resulting in an exclusion rate of 2.57%.

### 2.4 Data analysis

Data were analyzed using SPSS 27.0 and Mplus 8.3. Categorical variables were presented as frequencies and percentages. Continuous variables conforming to a normal distribution were expressed as mean ± standard deviation, while those not conforming to a normal distribution were presented as median and interquartile range. Group comparisons were performed using independent-sample *t*-tests or one-way ANOVA for normally distributed continuous variables, Mann–Whitney U tests or Kruskal–Wallis H tests for non-normally distributed continuous variables, and chi-square tests or Fisher's exact tests for categorical variables.

Latent profile analysis (LPA) was performed using Mplus 8.3 to identify distinct subgroups of digital health literacy among patients with coronary heart disease. Model fit was assessed using the following indicators: Akaike information criterion (AIC), Bayesian information criterion (BIC), sample-adjusted BIC (aBIC), entropy, Lo-Mendell-Rubin likelihood ratio test (LMR), and bootstrap likelihood ratio test (BLRT). Lower AIC, BIC, and aBIC values indicated better model fit. An entropy value closer to 1 indicated higher classification accuracy, with values ≥0.8 generally corresponding to classification accuracy exceeding 90%. Significant LMR and BLRT results (*P* < 0.05) indicated that a k-class model was significantly better than a k-1 class model (41).

All variables were entered into the final multinomial logistic regression model, with the most likely latent profile of digital health literacy as the dependent variable, to identify associated factors. A two-tailed P < 0.05 was considered statistically significant.

### 2.5 Ethical consideration

This study was conducted in accordance with the ethical guidelines of the Declaration of Helsinki. All eligible patients were provided with written informed consent prior to the survey, which included information about the study, the right to withdraw at any time, and confidentiality measures. Patient data were strictly confidential; data files were password-protected and accessible only to the research team to ensure privacy protection. The study was approved by the Medical Ethics Committee of the First Affiliated Hospital of Guangxi Medical University (Approval No. 2025-E0205).

## 3 Results

### 3.1 General information of the participants

A total of 350 questionnaires were distributed, and 341 valid questionnaires were returned, yielding an effective response rate of 97.43%. The mean age of the 341 patients with coronary heart disease was 63.38 ± 9.96 years. Additional demographic details are presented in Table 1.

**Table 1:** Univariate analysis of general characteristics and latent categories of digital health literacy among patients with coronary heart disease.

Variables		C1 *n* = 164 (48.1)	C2 *n* = 108 (31.7)	C3 *n* = 69 (20.2)	x^2^/F/H	*p*	Effect size
**Gender** *N* (%)	Male	103 (62.8)	85 (78.7)	59 (85.5)	15.648^(1)^	< 0.001	0.214^(4)^
	Female	61 (37.2)	23 (21.3)	10 (14.5)			
**Education level** *N* (%)	Junior high school or below	107 (65.2)	47 (43.5)	13 (18.8)	63.119^(1)^	< 0.001	0.304^(4)^
	High school or technical secondary school	34 (20.7)	27 (25)	16 (23.2)			
	Junior college	18 (11)	16 (14.8)	17 (24.6)			
	Bachelor's degree or above	5 (3)	18 (16.7)	23 (33.3)			
Marital status *N* (%)	Married	151 (92.1)	103 (95.4)	67 (97.1)	2.661^(1)^	0.264	0.088^(4)^
	Unmarried/divorced/widowed	13 (7.9)	5 (4.6)	2 (2.9)			
Medical insurance type *N* (%)	Employee medical insurance	27 (16.5)	33 (30.6)	35 (50.7)	35.334^(1)^	< 0.001	0.228^(4)^
	Resident medical insurance	120 (73.2)	61 (56.5)	23 (33.3)			
	Self-paid	17 (10.4)	14 (13)	11 (15.9)			
Smoking history *N* (%)	Yes	46 (28)	39 (36.1)	28 (40.6)	4.073^(1)^	0.131	0.109^(4)^
	No	118 (72)	69 (63.9)	41 (59.4)			
Drinking history *N* (%)	Yes	44 (26.8)	43 (39.8)	19 (27.5)	5.634^(1)^	0.06	0.129^(4)^
	No	120 (73.2)	65 (60.2)	50 (72.5)			
Disease duration *N* (%)	< 1 year	77 (47)	57 (52.8)	23 (33.3)	7.314^(1)^	0.12	0.104^(4)^
	1–5 year	69 (42.1)	37 (34.3)	36 (52.2)			
	>5 year	18 (11)	14 (13)	10 (14.5)			
Family history *N* (%)	Yes	9(5.5)	13 (12)	11 (15.9)	7.079^(1)^	0.029	0.144^(4)^
	No	155 (94.5)	95 (88)	58 (84.1)			
Place of residence *N* (%)	Urban	40 (24.4)	41 (38)	47 (68.1)	49.708^(1)^	< 0.001	0.270^(4)^
	Town	23 (14)	25 (23.1)	9 (13)			
	Rural	101 (61.6)	42 (38.9)	13 (18.8)			
Per capita monthly household income *N* (%)	< 2,000 RMB	71 (43.3)	17 (15.7)	5 (7.2)	53.803^(1)^	< 0.001	0.281^(4)^
	2,000–3,999 RMB	37 (22.6)	34 (31.5)	13 (18.8)			
	4,000–5,999 RMB	32 (19.5)	31 (28.7)	22 (31.9)			
	>6,000 RMB	24 (14.6)	26 (24.1)	29 (42)			
Self-rated economic status *N* (%)	Good	101 (61.6)	35 (32.4)	9 (13)	61.183^(1)^	< 0.001	0.300^(4)^
	Average	40 (24.4)	48 (44.4)	28 (40.6)			
	Bad	23 (14)	25 (23.1)	32 (46.4)			
Self-rated health status *N* (%)	Good	21 (12.8)	12 (11.1)	18 (26.1)	18.053^(1)^	0.001	0.163^(4)^
	Average	43 (26.2)	46 (42.6)	24 (34.8)			
	Bad	100 (61)	50 (46.3)	27 (39.1)			
Regular checkups *N* (%)	Yes	45 (27.4)	44 (40.7)	46 (66.7)	31.336^(1)^	< 0.001	0.160^(4)^
	No	119 (72.6)	64 (59.3)	23 (33.3)			
Regular exercise *N* (%)	Yes	66 (40.2)	42 (38.9)	41 (59.4)	8.743^(1)^	0.013	0.303^(4)^
	No	98 (59.8)	66 (61.1)	28 (40.6)			
Age (years, Mean ± SD)	63.38 ± 9.958	66.72 ± 8.04	60.81 ± 10.376	59.36 ± 10.827	20.835^(2)^	< 0.001	0.110^(5)^
BMI [M (P25, P75)]	24.75 (22.53, 26.93)	24.44 (22.06, 26.64)	25.125 (23.213, 27.138)	24.97 (22.42, 27.32)	2.23^(3)^	0.328	0.004^(5)^
Number of comorbidities [M (P25, P75)]	2 (2, 4)	2 (2, 4)	2 (1, 4)	3 (2, 4)	0.927^(3)^	0.629	0.014^(5)^
Number of co-residents [M (P25, P75)]	3 (2, 5)	4 (2, 6)	4 (2, 5)	3 (2, 4)	4.707^(3)^	0.095	0.003^(5)^
Total score of health belief [M (P25, P75)]	141 (127, 154)	132 (122, 143)	143 (131.25, 152)	158 (152, 167)	94.034^(3)^	< 0.001	0.277^(5)^
Total score of social support [M (P25, P75)]	41 (36, 46)	38 (34, 42)	43 (38, 46)	47 (42, 51)	77.414^(3)^	< 0.001	0.228^(5)^

(1) χ^2^ test; (2) F value; (3) H value; (4) Cramer's V; (5) η^2^.

### 3.2 Digital health literacy scale scores

The total score of the Digital Health Literacy Scale among the 341 patients with coronary heart disease was 19.27 ± 9.29, indicating a relatively low level. The scores for the three dimensions were as follows: application ability, 13.03 ± 6.23; evaluation ability, 4.08 ± 2.22; and decision-making ability, 2.15 ± 1.22. Item-level scores are presented in Table 2.

**Table 2:** Scores of the digital health literacy scale.

Variables	Mean	SD
Total score of digital health literacy	19.27	9.291
Application ability of online health information and services	13.03	6.23
D1	3.01	1.241
D2	2.63	1.348
D3	2.45	1.311
D4	2.48	1.33
D5	2.45	1.342
Critical thinking ability	4.08	2.22
D6	2.1	1.172
D7	1.98	1.135
Decision-making ability	2.15	1.22
D8	2.15	1.218

Table 2. Digital Health Literacy, Health Beliefs, and Social Support Scale Scores in CHD Patients (Mean ± SD).

### 3.3 Identification of latent DHL profiles in patients with CHD

Using the 8 items of the Digital Health Literacy Scale as indicators, LPA was conducted with Mplus 8.3. Models with one to four classes were evaluated, and the fit indices for each model are presented in Table 3. As the number of classes increased, the AIC, BIC, and aBIC values gradually decreased. The decline leveled off when moving from the three-class model to the four-class model. The entropy values for all models exceeded the critical value of 0.8, indicating good classification quality. In the three-class model, entropy reached its maximum value (0.980), and both the LMRT and BLRT were statistically significant (*p* < 0.001). The average posterior probabilities for the three classes were 0.996 (C1), 0.988 (C2), and 0.985 (C3), all far above the recommended threshold of 0.8. However, in the four-class model, entropy decreased to 0.958, and the LMRT result was no longer statistically significant (p = 0.255), indicating that the four-class model did not show a statistically significant improvement over the three-class model. Considering the goodness-of-fit indices and model parsimony, the three-class model was selected as the best-fitting model (see Table 3).

**Table 3:** Fit indices for latent DHL profiles in patients with CHD.

Class	AIC	BIC	aBIC	Entropy	LMR (*P*)	BLRT (*P*)	Proportion
1	9,025.537	9,086.847	9,036.092	–	–	–	–
2	6,689.081	6,784.878	6,705.573	0.958	0.0014	0.0016	0.54545/0.45455
3	5,659.422	5,789.706	5,681.851	0.980	< 0.001	< 0.001	0.48094/0.31672/0.20235
4	5,279.980	5,444.751	5,308.346	0.958	0.2553	< 0.001	0.32845/0.21701/0.26393/0.19062

**Figure 1:**
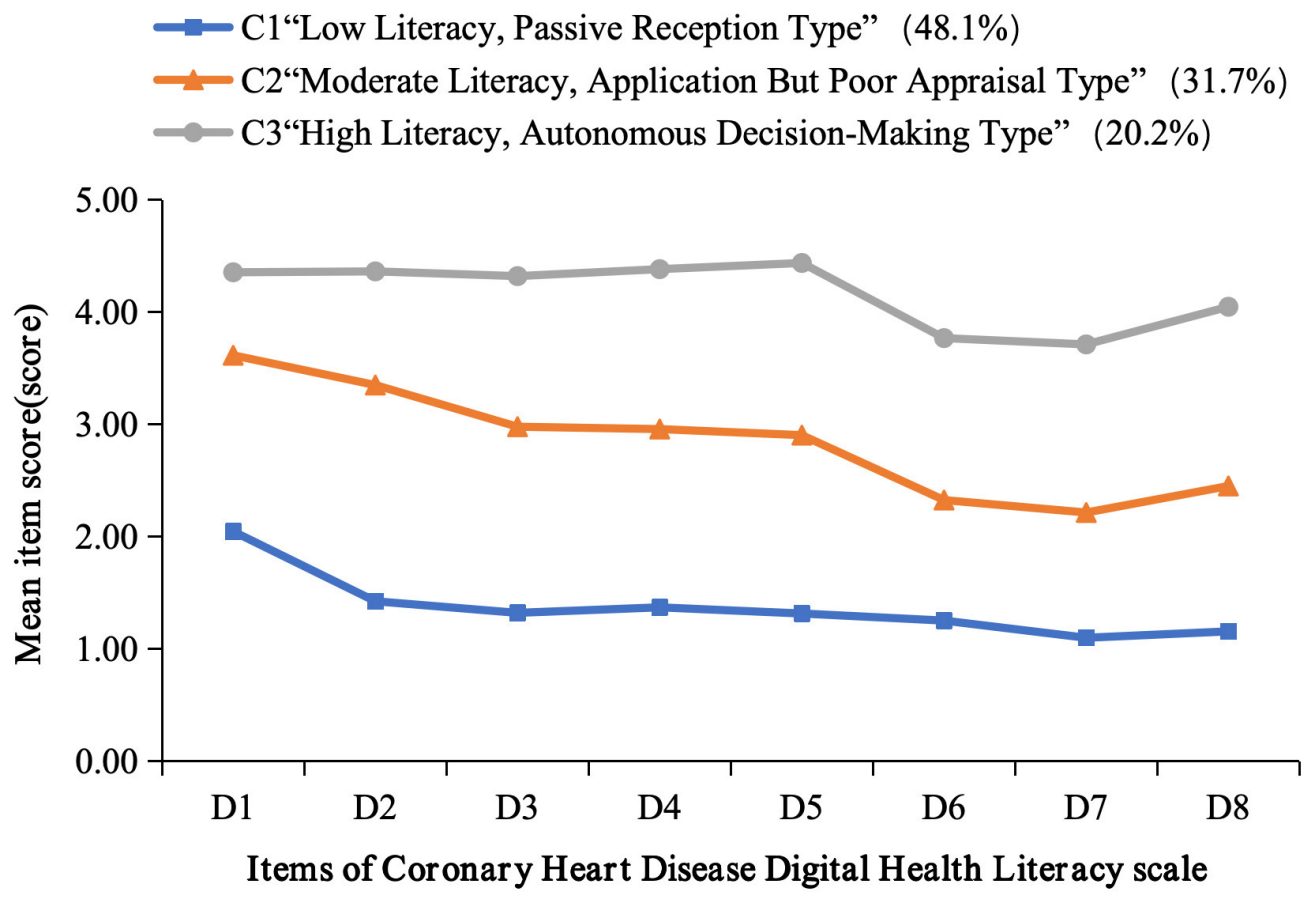
Latent profile chart of digital health literacy among patients with coronary heart disease.

Based on the three-class model, the mean scores of the eight digital health literacy items for each latent profile are presented in Figure 1. The three latent profiles of digital health literacy exhibited distinct patterns across the eight items, with minimal overlap (posterior probabilities > 0.98). They were named based on their characteristic score patterns.

C1 (*n* = 164, 48.1%) represented the largest proportion. Their mean scores on all eight items were the lowest among the three profiles, particularly on items 6–7 (critical evaluation) and item 8 (decision-making). Scores on item 1 (knowing how to find health resources) were relatively higher within this class, but still far below those of the other two profiles. Regarding the three dimensions, application ability (items 1–5) was very low, critical evaluation (items 6–7) was extremely poor, and decision-making (item 8) was severely deficient. This group demonstrated overall low digital health literacy and tended to receive health information passively rather than actively seeking or evaluating it. This profile was therefore named the “Low Literacy, Passive Reception Type”.

C2 (*n* = 108, 31.7%) was the second-largest proportion. Their mean scores on items 1–5 (application ability) were moderate, but scores on items 6–7 (critical evaluation) and item 8 (decision-making) remained low. Scores gradually decreased from item 1 to item 3 and leveled off between items 3 and 4. In terms of the three dimensions, application ability was moderate, but critical evaluation and decision-making showed marked deficits. Consequently, their health decisions may still rely on others' advice. This profile demonstrated moderate digital health literacy overall, but with a clear bottleneck in critical appraisal and decision-making. This profile was therefore named the “Moderate Literacy, Application but Poor Appraisal Type”.

C3 (*n* = 69, 20.2%) represented the smallest proportion. Their mean scores on all eight items were the highest, especially on items 1–5 (application ability) and item 8 (decision-making), with the highest score observed on item 5 (using information to help oneself). Scores on items 6 and 7 (critical evaluation) were also high. Across the three dimensions, application ability, critical evaluation, and decision-making were all well developed. This indicates that these patients could independently integrate and apply online health information for self-management and possessed strong decision-making capacity. This profile demonstrated high digital health literacy with autonomous decision-making. This profile was therefore named the “High Literacy, Autonomous Decision-Making Type”.

### 3.4 Univariate analysis of digital health literacy latent profiles among patients with coronary heart disease

Univariate analysis revealed no statistically significant differences in the distribution of BMI, smoking history, alcohol consumption history, disease duration, number of comorbidities, or number of people living together (all *P* > 0.05).

However, the three latent profiles showed statistically significant differences in terms of gender (χ^2^ = 15.648, *p* < 0.001), age (*F* = 20.835, *p* < 0.001), educational attainment (χ^2^ = 63.119, *p* < 0.001), type of medical insurance (χ^2^ = 35.334, *p* < 0.001), place of residence (χ^2^ = 49.708, *p* < 0.001), monthly income (χ^2^ = 53.803, *p* < 0.001), self-assessed economic status (χ^2^ = 61.183, *p* < 0.001), self-assessed physical condition (χ^2^ = 18.053, *p* < 0.001), regular physical examinations (χ^2^ = 31.336, *p* < 0.001), regular exercise (χ^2^ = 8.743, *p* = 0.013), and family history (χ^2^ = 7.079, *p* = 0.029) (all *P* < 0.05).

In addition, significant differences were observed in the total scores of health beliefs (H = 94.034, *p* < 0.001) and social support (H = 77.414, *p* < 0.001) among the three profiles (both *P* < 0.05). The health belief and social support scores were highest in C3, followed by C2, and lowest in C1 (see Table 1).

### 3.5 Multinomial logistic regression of digital health literacy latent profiles among patients with coronary heart disease

Although the three latent profiles demonstrated an apparent ascending trend (low–moderate–high), they exhibited distinct item-level response patterns (Figure 1) rather than a simple ordinal hierarchy. Multinomial logistic regression was therefore employed to estimate profile-specific effects flexibly. All categorical variables were entered as independent variables, together with age, social support, and total health belief score as covariates. A multinomial logistic regression analysis was performed with the three-category latent profile (C1 = 1, C2 = 2, C3 = 3) as the dependent variable. Before regression analysis, multicollinearity among continuous variables was examined using the variance inflation factor (VIF); all VIF values were below 5 (age: 1.04; social support: 1.48; health belief: 1.43), indicating no multicollinearity. Categorical variables were entered as factors in SPSS, which automatically performs dummy coding using the highest-coded category as the reference group (marked with ^*^ in Table 4). Detailed variable coding and assignment are shown in Table 4. To obtain pairwise comparisons among the three latent profiles, multinomial logistic regression was conducted twice: first using C1 as the reference category, and then using C3 as the reference category. Results from the two models were consistent. Odds ratios for C2 vs. C1, C3 vs. C1, and C2 vs. C3 are reported in Table 5.

**Table 4:** Variable coding scheme.

Variable	Coding
Genders	Male = 1, Female = 2^*^
Education level	Junior high school or below = 1, high school or technical secondary school = 2, junior college = 3, bachelor's degree or above = 4^*^
Marital status	Married = 1, unmarried/divorced/widowed = 2^*^
Medical insurance type	Employee medical insurance = 1, resident medical insurance = 2, self-paid = 3^*^
Smoking history	Yes = 1, No = 2^*^
Drinking history	Yes = 1, No = 2^*^
Disease duration	< 1 year = 1, 1–5 year = 2, >5 year^*^
Family history	Yes = 1, no = 2^*^
Place of residence	Urban = 1, town = 2, rural = 3^*^
Per capita monthly household income	< 2,000 RMB = 1, 2,000–3,999 RMB = 2, 4,000–6,000 RMB = 3, >6,000 RMB = 4^*^
Self-rated economic status	Good = 1, average = 2, bad = 3^*^
Self-rated health status	Good = 1, average = 2, bad = 3^*^
Regular checkups	Yes = 1, no = 2^*^
Regular exercise	Yes = 1, no = 2^*^
Age, BMI, number of comorbidities, number of people living together, total score of health belief, total score of social support	Original value input

^*^Indicates the reference category.

**Table 5:** Multivariate analysis of digital health literacy latent profiles among patients with coronary heart disease (*n* = 341).

Variables	β	SE	Wald *x*^2^	*p*	OR	95% CI	
						Lower limit	Upper limit
C2 vs. C1^*^							
Intercept	−2.549	3.104	0.674	0.412			
Age	−0.117	0.023	25.140	< 0.001	0.89	0.850	0.931
Junior high school or below	−2.417	0.750	10.394	0.001	0.089	0.021	0.388
High school or technical secondary school	−1.972	0.756	6.796	0.009	0.139	0.032	0.613
Junior college	−2.486	0.830	8.963	0.003	0.083	0.016	0.424
15.6-7.4,-1.3498pt Total score of social support	0.141	0.031	20.081	< 0.001	1.151	1.082	1.224
C3 vs. C1^*^							
Intercept	−5.373	4.625	1.350	0.245			
Age	−0.186	0.032	33.965	< 0.001	0.830	0.779	0.884
Junior high school or below	−3.325	0.904	13.533	< 0.001	0.036	0.006	0.211
High school or technical secondary school	−2.120	0.868	5.966	0.015	0.120	0.022	0.658
Junior college	−2.397	0.938	6.524	0.011	0.091	0.014	0.573
Urban	1.547	0.684	5.109	0.024	4.496	1.228	17.955
Total score of social support	0.116	0.042	7.637	0.006	1.123	1.034	1.220
15.6-7.4,-1.3498pt Total score of health belief	0.091	0.020	19.944	< 0.001	1.095	1.052	1.139
C2 vs. C3^*^							
Intercept	7.921	4.290	3.410	0.065			
Age	0.070	0.026	6.895	0.009	1.072	1.018	1.129
Total score of health belief	−0.080	0.018	19.811	< 0.001	0.923	0.891	0.956

^*^For the reference group; the highest assignment was used as the control for all independent variables. OR, odds ratio; CI, confidence interval; LLCI, lower limit of confidence interval; ULCI, upper limit of confidence interval. *P* values indicate statistical significance.

Multinomial logistic regression analysis revealed that, with C1 as the reference, age (OR = 0.89, 95% CI: 0.850–0.931, *p* < 0.001) and social support (OR = 1.151, 95% CI: 1.082–1.224, *p* < 0.001) were independent factors associated with C2. Specifically, younger age and higher levels of social support were associated with a greater likelihood of belonging to C2. In practical terms, each one-year increase in age reduced the odds of being in C2 by 11%, while a one-point increase in social support score increased the odds by 15%. Compared with patients with a bachelor's degree or above, those with junior high school or below (OR = 0.089, 95% CI: 0.021–0.388, *p* = 0.001), high school or technical secondary school (OR = 0.139, 95% CI: 0.032–0.613, *p* = 0.009), and junior college education (OR = 0.083, 95% CI: 0.016–0.424, *p* = 0.003) were significantly less likely to be classified into C2. Patients with a bachelor's degree or higher had more than 10-fold higher odds of being in C2 than those with junior high school or below.

In the comparison between C3 and C1, age (OR = 0.830, 95% CI: 0.779–0.884, *p* < 0.001), place of residence (OR = 4.496, 95% CI: 1.228–17.955, *p* = 0.024), social support (OR = 1.123, 95% CI: 1.034–1.220, *p* = 0.006), and health beliefs (OR = 1.095, 95% CI: 1.052–1.139, *p* < 0.001) were identified as independent factors associated with C3. Specifically, younger age, urban residence, higher levels of social support, and stronger health beliefs were associated with a greater likelihood of belonging to C3. Notably, urban residents were 4.5 times more likely to be in C3 than rural residents. A one-point increase in health belief score increased the odds by approximately 10%. Compared with patients with a bachelor's degree or above, those with junior high school or below (OR = 0.036, 95% CI: 0.006–0.211, *p* < 0.001), high school or technical secondary school (OR = 0.120, 95% CI: 0.022–0.658, p = 0.015), and junior college education (OR = 0.091, 95% CI: 0.014–0.573, *p* = 0.011) were significantly less likely to be classified into C3. Having a bachelor's degree or above increased the odds of being in C3 by nearly 28-fold compared to junior high school or below.

In the comparison between C2 and C3, health belief (OR = 0.923, 95% CI: 0.891–0.956, *p* < 0.001) and age (OR = 1.072, 95% CI: 1.018–1.129, *p* = 0.009) were identified as independent factors associated with C2. Specifically, patients with lower health belief scores and older age were more likely to be in C2 than in C3. A one-point increase in health belief score was associated with 8% higher odds of being in C3 vs. C2.

In conclusion, age, educational level, place of residence, social support, and health beliefs were identified as significant factors associated with the latent profiles of digital health literacy among patients with coronary heart disease (all *p* < 0.05), as detailed in Table 5.

## 4 Discussion

### 4.1 Digital health literacy among patients with coronary heart disease was generally low

In this study, the digital health literacy score of patients with coronary heart disease was 19.27 ± 9.29, indicating a relatively low level. This finding aligns with previous studies showing low digital health literacy in Chinese chronic disease populations, but the observed score was lower than that reported by Shao et al. (42) (41.36 ± 12.8) and Jia et al. (27) (32.17 ± 14.51). The difference may be attributable to the characteristics of the study population. Participants in this study were recruited from a single hospital, which may limit the generalizability of the findings. Only 37.5% of the participants were from urban areas, suggesting that digital health literacy among patients with coronary heart disease in rural and town areas may be lower than that of their urban counterparts. Additionally, all participants were inpatients. It is possible that during hospitalization, they received substantial health information from healthcare providers, which may have reduced their attention to digital health resources. Research has shown that older adults and individuals with lower educational levels have weaker abilities to access digital information and use digital devices (9). Nearly half of the participants (49%) had a junior high school education or below, which may further contribute to lower digital health literacy. These findings are consistent with previous studies, indicating that age, place of residence, and educational level are key associated factors (9). These factors may explain the generally low level of digital health literacy observed among participants in this study.

### 4.2 Digital health literacy among patients with coronary heart disease can be categorized into three latent profiles

In this study, digital health literacy among patients with coronary heart disease was classified into three latent profiles: C1 (Low Literacy, Passive Reception Type), C2 (Moderate Literacy, Application but Poor Appraisal Type), and C3 (High Literacy, Autonomous Decision-Making Type). This suggests significant heterogeneity in digital health literacy within this population.

C1 (*n* = 164, 48.1%) had the largest proportion in this study, and its overall digital health literacy was relatively low. Most of these patients were from rural areas and had a junior high school education or below. Their mean age was 66.72 ± 8.04 years, which was higher than that of the other profiles. In addition, they had the lowest scores in social support and health beliefs, and their mean scores on each digital health literacy item were significantly lower than those of the other profiles. This profile generally appeared to lack the ability to effectively search for, identify, and apply online health information. These characteristics may be closely related to factors such as advanced age, lower educational level, rural residence, insufficient health beliefs, and lack of social support.

Patients in C2 (*n* = 108, 31.7%) had moderate digital health literacy. Their scores on each item were average, but their scores on the judgment and decision-making dimensions were relatively low, suggesting that although these patients possess basic information-seeking skills, they lack confidence in evaluating and applying information. Consequently, they still rely on others' advice when making health decisions. In this profile, those who self-assessed their economic status as “average” accounted for the largest proportion. The rate of regular physical examinations was 40.7%, higher than in C1 (27.4%). The proportion of participants with a bachelor's degree or higher was 16.7%, a substantial increase from C1 (3.0%). Similarly, the proportion covered by employee medical insurance was 30.6%, also higher than that in C1 (16.5%). Both health beliefs and social support were at moderate levels. These characteristics suggest that among patients in C2, those with higher education levels and employee medical insurance were more prevalent in this profile, were more aware of self-health management, and possessed better social support resources.

C3 (*n* = 69, 20.2%) represented the smallest proportion in this study, suggesting that patients with coronary heart disease who have high digital health literacy remain a minority. This profile had the highest mean scores on all digital health literacy items, with the highest score on the item “knowing how to use online health information resources to help oneself.” However, their scores on the evaluation ability dimension remained relatively low. Patients in C3 had the following characteristics: their mean age was 59.36 ± 10.83 years, which was lower than that of C1 and C2; they had the highest proportions of bachelor's degree or above (33.3%), employee medical insurance coverage (50.7%), and urban residence (68.1%); they also had the highest rates of regular physical examinations and regular exercise among the three groups. Both social support and health belief scores were the highest among the three groups, indicating that this group possesses favorable psychological resources and a supportive network. In terms of digital health capabilities, C3 patients were proficient with smart devices and various health resources, were able to critically evaluate online information, and demonstrated a strong sense of self-health management.

### 4.3 The latent profiles of digital health literacy among patients with coronary heart disease are influenced by multiple factors

#### 4.3.1 Individual-level factors (age, education level, place of residence)

In our study, older age was associated with lower digital health literacy, consistent with previous evidence of a negative correlation between age and digital health literacy (7, 43). Specifically, each one-year increase in age reduced the odds of belonging to the C2 or C3 profile by approximately 11%−17%. This association may reflect age-related declines in physical function, cognitive level, and reaction ability, which reduce individuals' perception of online health information resources, ultimately hindering improvements in digital health literacy (26, 43). Older patients tend to have a weaker ability to adopt new online practices, may be more dependent on traditional medical channels, and lack the motivation to actively explore digital health resources. Consequently, older age was associated with a higher likelihood of belonging to the C1 profile.

Lower educational level was associated with lower digital health literacy, consistent with previous findings (7, 44). Individuals with higher levels of education are better able to understand and evaluate the credibility of online health information and to effectively obtain and apply medical and health information. Lower education level, insufficient literacy, limited exposure to digital media, and limited device proficiency are factors associated with lower digital health literacy (44).

Urban residence was associated with higher digital health literacy, with urban patients being 4.5 times more likely to belong to C3 than rural residents. This finding aligns with studies showing that economic development is positively correlated with digital health literacy (28, 45). In more economically developed regions, residents generally have higher levels of digital health literacy. However, findings regarding urban-rural differences remain inconsistent; for example, Zuo et al. (46) found rural residents had higher eHEALS scores than urban residents in Chengdu. Such inconsistencies may be attributed to the fact that most existing studies rely on self-reported data, which reflect individuals' subjective perceptions and may be subject to measurement bias. A study of patients with heart failure in the United States also found that access to mobile health technologies was closely associated with age, rural residence, and socioeconomic factors (45).

#### 4.3.2 Social support

Higher levels of social support were associated with greater odds of belonging to C2 or C3 rather than C1. Specifically, each one-point increase in social support score increased the odds of being in C2 by 15% and in C3 by 12%. These patients typically possess good health management skills, can communicate effectively with healthcare providers, and obtain professional medical information in a timely manner, thereby reducing barriers to information access. Meanwhile, the diverse social support they receive-emotional, informational, and material-provides a strong foundation for proactive and efficient health management. Such patients tend to be more proactive in seeking health information and actively engage in interactions on digital health platforms (47). Adequate social support is associated with better self-management ability and treatment adherence. Family members sharing health-related knowledge through digital media, such as WeChat and health websites, may play a positive role in improving individuals' digital health literacy (48). Peer support also appears important; Information shared within peer networks is often perceived as more authentic and reliable. Such interactions may have both guiding and motivating effects (49) that could promote improvements in digital health literacy among patients with coronary heart disease. Therefore, improving digital health literacy is related not only to individuals' health management abilities but also to diverse support networks involving family, peers, and society. Future efforts could further explore the development of a “family-peer-hospital” interactive support system.

#### 4.3.3 Health beliefs

Stronger health beliefs were associated with higher digital health literacy, particularly in the comparison between C3 and C1 (OR = 1.10 per point) and between C3 and C2 (OR = 1.08 per point). Previous studies also support this view. A recent meta-analysis found that subjective norms, technology self-efficacy, perceived usefulness, and attitude were strong predictors of consumers' willingness to use health technologies (50). Zhou et al. (51) found that digital media is associated with enhanced individuals' health cognition, which may strengthen health management beliefs and lay a cognitive foundation for improving digital health literacy. Ghazi et al. (52) also reported that higher DHL is associated with better self-rated health status and mental health, which indirectly reflects the positive association between positive health beliefs and digital health literacy.

Among patients with coronary heart disease, those with higher levels of health beliefs typically have a clearer understanding of their disease risks and a deeper appreciation of the value of active health management, and are therefore more inclined to adopt positive health behaviors. Against the backdrop of rapidly developing digital healthcare, this intrinsic motivation naturally extends to the digital health domain. Such patients are more willing to try health-related applications, actively search for authoritative medical information, participate in online doctor-patient interactions, and gradually accumulate digital health skills, thereby enhancing their digital health literacy through practice.

It is worth noting that, in this study, health beliefs reached statistical significance only in the comparisons between C3 and C1 and between C2 and C3, but not between C2 and C1. This finding suggests that health beliefs may be a key psychological factor enabling individuals to transition from low to high levels of digital health literacy.

Against the backdrop of rapidly advancing smart healthcare and digital health services, enhancing digital health literacy among patients with coronary heart disease has become essential for disease prevention and proactive health management. Special attention should be paid to older adults, rural residents, and patients with lower education levels. It is recommended to strengthen their health beliefs, improve social support systems, develop personalized health education content, and encourage family involvement to promote digital health literacy among these patients (8). Patients' digital health literacy can be improved by building a multi-agent collaborative social support network. For example, the government can strengthen patients' awareness of digital health and their health beliefs through policy guidance and public awareness campaigns (53). Enterprises should promote the research, development, and dissemination of age-friendly technologies to lower the barriers for patients (5). Communities should optimize health services, build support networks, and strengthen patients' willingness to use services and their health beliefs (54, 55). Relying on professional medical guidance, hospitals should enhance patients' digital skills and health awareness through positive interaction (8), encourage families to provide emotional and practical support, and help patients establish a sense of health responsibility and take initiative in their own care.

## 5 Limitations

This study has the following limitations. First, the survey population was recruited solely from patients with coronary heart disease in a tertiary Grade A hospital in Guangxi, China. This may limit the generalizability of the findings to patients in other regions or those treated in primary and secondary healthcare settings. Consequently, the results may lack broad representativeness. Second, data were collected within 24–48 h of hospitalization. Although patients were clinically stable at the time of assessment, this early stage may still have influenced self-reported responses. Third, all data were collected via self-report questionnaires, which may introduce common method biases, including social desirability and recall biases. Fourth, although the classification accuracy of the latent profile analysis was relatively high (entropy >0.8), classification uncertainty was not adjusted for in the regression model. Future studies may adopt more appropriate bias correction methods to improve estimation precision. Finally, this study adopted a cross-sectional, one-off survey design and therefore could not examine causal relationships among the variables. In the future, multicenter, large-sample longitudinal studies should be considered to further clarify the potential causal relationships and pathways of influence among digital health literacy, social support, health beliefs, and their associated factors in patients with coronary heart disease.

## 6 Conclusion

This study found that DHL among patients with CHD was relatively low. LPA identified three distinct profiles: Low Literacy, Passive Reception Type (C1, 48.1%); Moderate Literacy, Application But Poor Appraisal Type (C2, 31.7%); and High Literacy, Autonomous Decision-Making Type (C3, 20.2%). There were significant differences across the three profiles. Healthcare providers should prioritize the C1 profile while also attending to the C2 profile. Personalized interventions tailored to the characteristics of each profile are needed to improve DHL, disease prognosis, and quality of life in this population.

## Data Availability

The raw data supporting the conclusions of this article will be made available by the authors, without undue reservation.
